# Chromophore Renewal and Fluorogen-Binding Tags: A Match Made to Last

**DOI:** 10.1038/s41598-017-12400-9

**Published:** 2017-09-26

**Authors:** Frederico M. Pimenta, Giovanni Chiappetta, Thomas Le Saux, Joëlle Vinh, Ludovic Jullien, Arnaud Gautier

**Affiliations:** 1 École Normale Supérieure, PSL Research University, UPMC Univ Paris 06, CNRS, Département de Chimie, PASTEUR, 24 rue Lhomond, 75005 Paris, France; 2 Sorbonne Universités, UPMC Univ Paris 06, ENS, CNRS, PASTEUR, 75005 Paris, France; 3 ESPCI Biological Mass Spectrometry and Proteomics USR 3149 CNRS/ESPCI-PSL, Paris, France

## Abstract

Fluorogen-binding tags, which activate the fluorescence of a specific chromophore (so-called fluorogen) upon reversible binding, have recently been proposed as a way of reducing photobleaching *via* fluorogen renewal. However, no generic methodology has been proposed to systematically analyze the photodamage of the fluorogen and the protein tag. Using Y-FAST (Yellow Fluorescence-activating and Absorption-Shifting Tag) as a case study we propose here a generic experimental and theoretical approach to assess how fluorogen renewal reduces the apparent photobleaching rate of a fluorogen-binding tag. Y-FAST has its apparent photobleaching rate greatly reduced by fluorogen renewal and its photostability is mainly limited by oxidation of specific residues in the protein scaffold by reactive oxygen species generated by the bound fluorogen. This study sets the groundwork for the optimization of fluorogenic systems, helping guide rational improvements to their photostability.

## Introduction

Microscopy has been the technique of choice for probing and answering important biological questions for over a century. In addition to the advances in optics, detectors and different microscopy techniques^1–3^, biomolecular imaging has benefited from the discovery of the Green Fluorescent Protein (GFP)^4^ and other FPs^1^, which provide labelling specificity in living cells and animals. Continuous development of several protein-based biosensors has allowed the scientific community to answer relevant biological questions in areas as diverse as protein localization and concentration changes^5^, protein-protein interactions^6^, and ion and organic molecule fluxes^7^. Despite all these advances, photobleaching of fluorescent probes and biosensors is still a recurring issue in most imaging techniques. Several strategies can be employed to minimize photobleaching (e.g. chemical modifications of the chromophore^8^ or the use of antioxidants and triplet excited state quenchers during live cell imaging)^9,10^, but these can interfere with the properties one wants to measure and/or be inapplicable to certain systems (e.g. most changes to the chromophore of a typical FP will result in fluorescence loss/change)^2^.

Photobleaching encompasses a plethora of photophysical and photochemical phenomena that result in an irreversible loss of fluorescence. These processes largely depend on the photophysical, photochemical, and redox properties of the chromophore, its immediate surrounding environment and can arise from processes as distinct as molecular rearrangements or oxygen addition^11^. In certain FPs, processes such as covalent bond formation between the protein scaffold and the chromophore have also been shown to lead to photobleaching. The photostability of FPs is still one order of magnitude below certain organic dyes^12^ and significant engineering steps are being taken to characterize^13–19^ and rationally improve their photostability^20^. However, typical FPs are limited to a single chromophore that once bleached can no longer be replaced. Conversely, fluorogen-based reporters, composed of a genetically-encoded biomolecule (e.g., protein or RNA aptamer) that activates the fluorescence of a chromophore (a so-called fluorogen) upon reversible binding, have recently been proposed as candidates to increase photostability^8,21–23^. Studies on the RNA-aptamer Spinach^23^ system, and some fluorogens^8^ of the Fluorogen-Activating Proteins (FAPs) series^24–27^ indicate that some of these systems have the ability to exchange bound fluorogens with new ones and avoid the accumulation of photodamaged reporters.

**Figure 1:**
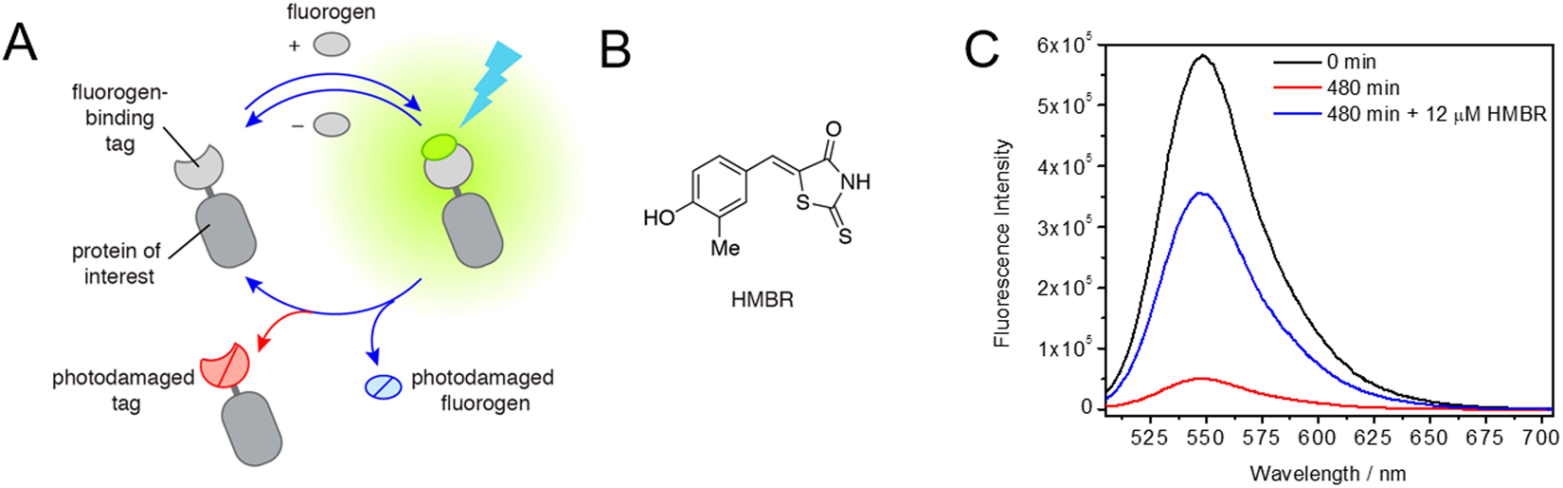
(**A**) Scheme depicting the active processes upon irradiation of a fluorogen-binding tag. In an ideal scenario (in blue), only the dye is photodamaged while in a real scenario observed in the present system, both the fluorogen (at a rate p_1_k_1B_) and the protein tag (at a rate [1–p_1_]k_1B_) are photodamaged during irradiation. (**B**) Structure of the fluorogen used in this study, 4-hydroxy-3-methylbenzylidene-rhodanine (HMBR). (**C**) Fluorescence emission spectra of an equimolar solution of Y-FAST:HMBR (3:3 μM, ∼80% complex formed) recorded as a function of the irradiation time at 488 nm (243 W m^−2^) and posterior addition of 12 μM of HMBR, demonstrating the principle of chromophore renewal.

To our knowledge, no study has so far described a general method correlating fluorogen renewal with reducing photobleaching and only a handful of examples have studied more in-depth the photobleaching mechanisms in specific fluorogenic systems^8,28^. To fill that gap we propose a general methodology to analyse: (i) whether and how fluorogen renewal reduces the apparent photobleaching rate of a given fluorogen-based reporter and (ii) what are the limiting factors in the photostability of fluorogen-based reporters. As a case study, we focused on a new fluorogen-based reporter named Yellow Fluorescence-activating and Absorption-Shifting Tag (Y-FAST), generated by directed evolution of the apo-form of the Photoactive Yellow Protein (PYP)^29^. Y-FAST reversibly binds a family of synthetic chromophores^29,30^, the most relevant for the present study being 4-hydroxy-3-methylbenzylidene-rhodanine (HMBR) (Fig. 1A,B). HMBR binding to Y-FAST, *via* deprotonation of the phenolate moiety, induces an absorption red-shift (∼80 nm) at physiological pH which, combined with a fluorescence quantum yield increase of over a 1000 times (corrected here to ϕ_F_ = 0.23 ± 0.03, see Figure S1 and SI Text 1), renders this chromophore essentially dark unless bound to its protein partner. Y-FAST differs from other fluorogen-based systems in its fast and reversible binding of HMBR (dissociation constant *K*_D_ of ∼0.1 μM, and a residence time in the binding pocket of ∼160 ms), making fluorogen renewal an *a priori* extremely fast process.

## Results and Discussion

**Figure 2:**
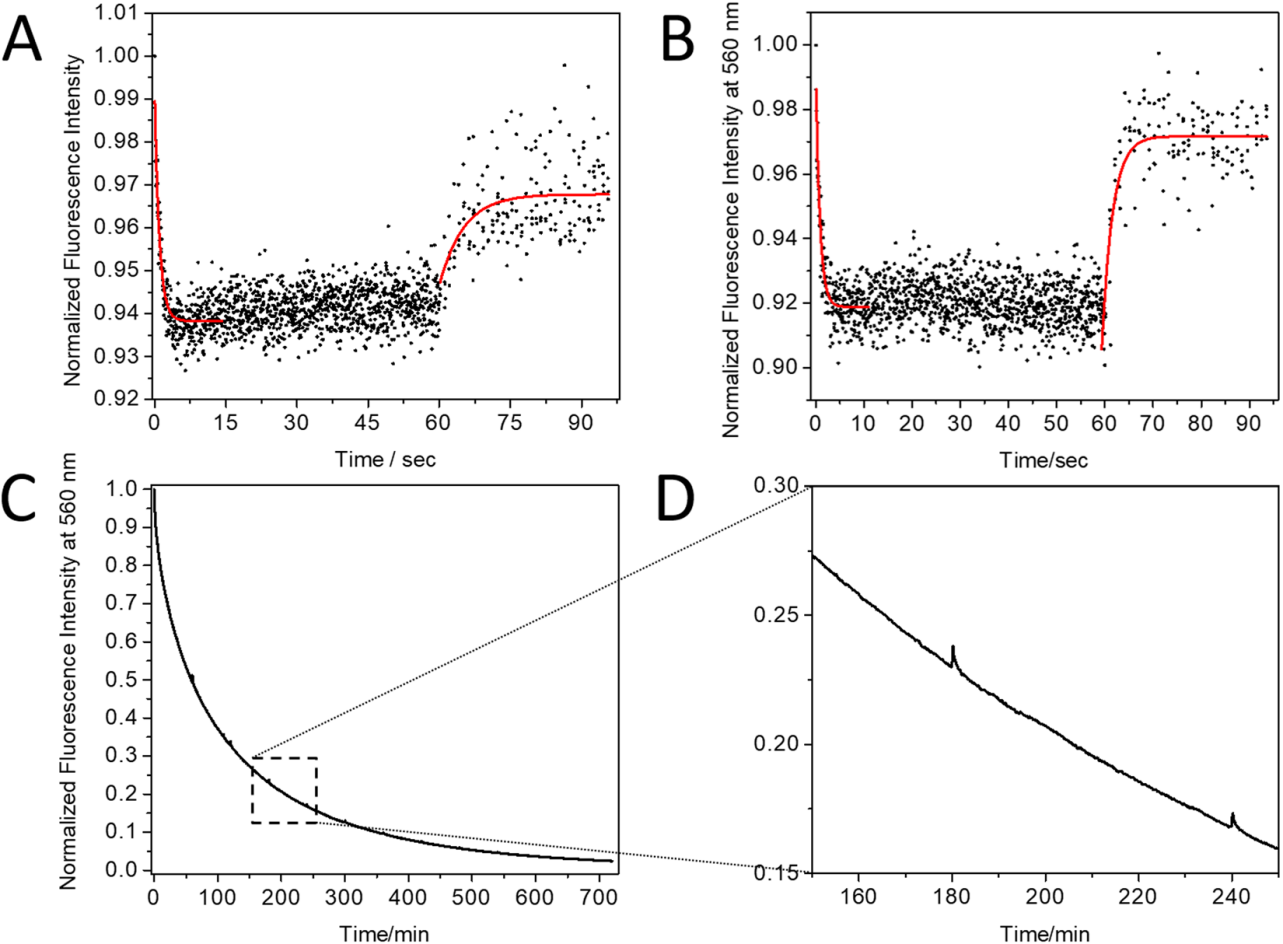
(**A**) Plot of the normalized fluorescence intensity of a 3.7 µM HMBR solution (pH 10.5) at 560 nm upon continuous irradiation at 488 nm (71 W m^−2^) and subsequent relaxation in darkness. The observed photoconversion is characterized by a relaxation time of 0.9 s and a thermal relaxation time of 4.5 s. (**B**) Plot of the normalized fluorescence intensity of a solution of the Y-FAST:HMBR (15:4 µM) complex at 560 nm upon continuous irradiation at 488 nm (71 W m^−2^) and subsequent relaxation in darkness. The observed photoconversion is characterized by a relaxation time of 1.1 s and a thermal relaxation time of 1.5 s. (**C**) Plot of the normalized fluorescence intensity of the Y-FAST:HMBR complex (15:3 µM) at 560 nm as a function of elapsed irradiation time at 488 nm (243 W m^−2^). Irradiation was ceased for 5 minutes at every hour, resulting in a small dark state conversion highlighted in (**D**) (See SI Text S2)

To study the contribution of fluorogen renewal to photostability, we first measured the changes in fluorescence of Y-FAST in solution under irradiation with 488 nm (light intensity 243 W m^−2^) for 8 hours, which lead to an extensive and irreversible loss of fluorescence. Addition of fresh HMBR restored most of the initial fluorescence (∼60%), indicating an efficient exchange of generated photoproducts with unbleached HMBR (Fig. 1C). Under low light intensities, we also observed a small reversible loss of fluorescence likely due to fluorogen photoisomerization (Fig. 2A,B). This phenomenon plays however a minor role in the overall loss of fluorescence (Fig. 2C,D and SI Text 2).

**Figure 3:**
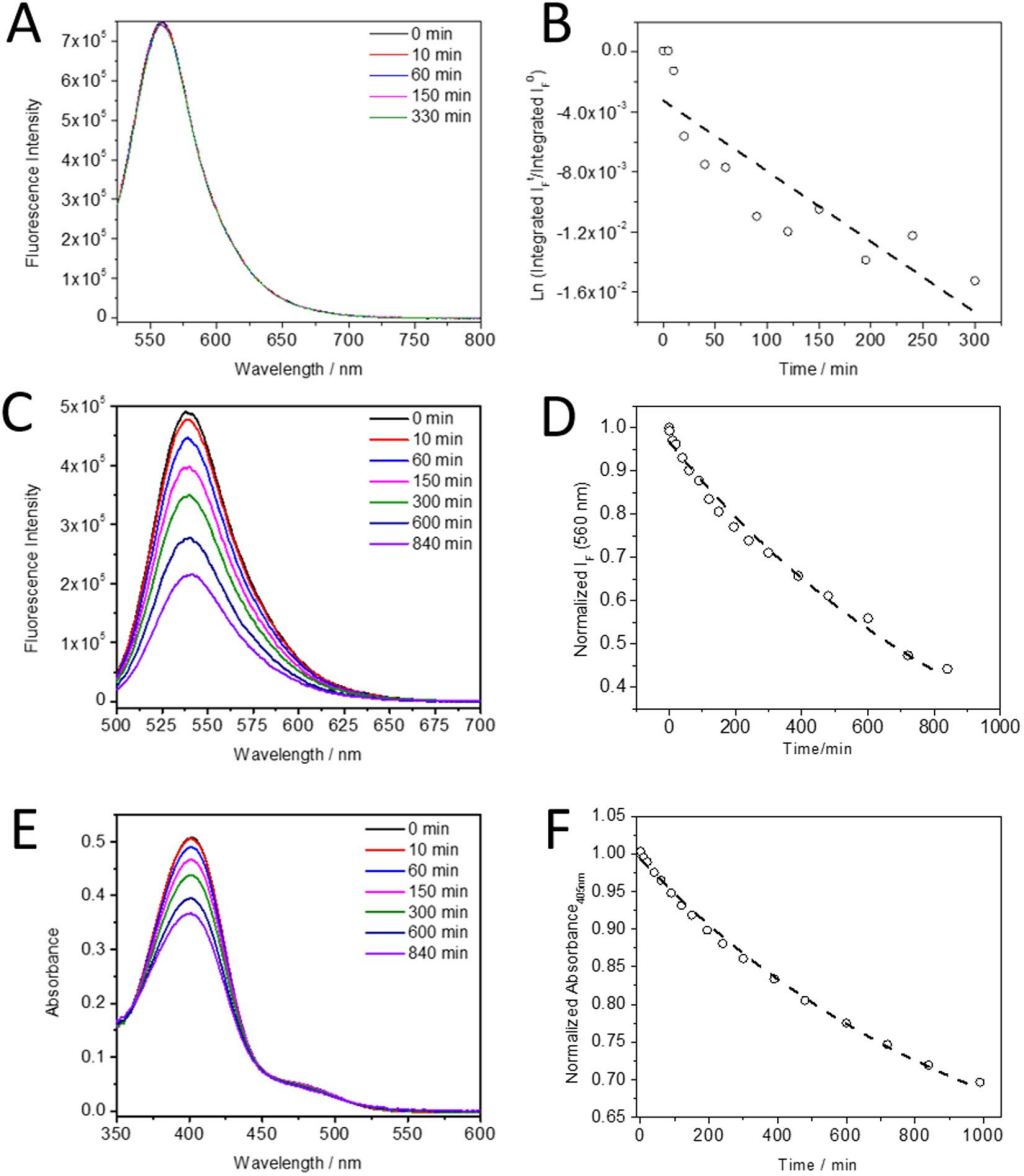
(**A**) Selected fluorescence emission spectra of a 15 µM HMBR solution in PBS pH 7.4 recorded as a function of irradiation at 488 nm (324 W m^−2^). (**B**) Neperian logarithm plot of the normalized integrated fluorescence intensity of HMBR bleaching over time used to extract the photobleaching rate constant of HMBR free in solution ($\mathrm{Ln}\int {I}_{F}/\int {I}_{F}^{0}=-{k}_{F}\times t)$, yielding ${k}_{\text{F}}=$(4.7 ± 0.8) × 10^−5^ min^−1^. (**C**) Selected fluorescence emission and (**E**) absorption spectra of Y-FAST:HMBR (0.4:15 µM, ~100% complex formed during the entire experimental time window) solvated in PBS pH 7.4, recorded as a function of irradiation at 488 nm. (**D**) Plot of the normalized fluorescence intensity of the Y-FAST:HMBR at 560 nm as a function of irradiation time, with a single exponential fit applied to the data, used to determine k_P_, (9.9 ± 0.3) × 10^−4^ min^−1^. (**F**) Plot of the normalized absorbance of the Y-FAST:HMBR at 405 nm as a function of irradiation time, where a single exponential fit applied to the data was used to determine p_1_ and k_1B_ (pre-exponential factor A^405 nm^ = 0.489 ± 0.008).

We next quantified the individual photobleaching rates of the protein tag and the fluorogen. Solutions of HMBR and Y-FAST:HMBR (in large excess of HMBR) in PBS at pH 7.4 were irradiated at 488 nm (324 W m^−2^) and the temporal evolutions of the absorbance and fluorescence emission were analysed with a theoretical framework that encompassed all possible reactions for the Y-FAST:HMBR complex, i.e., fluorogen exchange, fluorogen photoisomerization, and photodestruction of either the tag or the fluorogen (the latter either free or bound to Y-FAST) (see SI Text 3). Note that the mathematical model accounts for the photodestruction of the tag and the fluorogen without assuming *a priori* any specific photodestruction mechanisms. The complete reactive scheme was reduced to a simpler kinetic model by taking into account that fluorogen exchange and fluorogen photoisomerization occur at a much shorter time scale than the observed photodestruction reactions (seconds as opposed to hours). Hence we could use the fluorescence decays to show that: (i) the fluorogen bleaching upon 488 nm excitation in the absence of Y-FAST (Fig. 3A,B) is three orders of magnitude slower than in its presence; (ii) the photodamage of the protein tag occurred with a rate constant (*k*_*P*_) of (9.9 ± 0.3) × 10^−4^ min^−1^ (Fig. 3C,D, and SI Text 3). The latter was then used to fit the absorption changes over time with irradiation (Fig. 3E,F), providing the photobleaching rate constant for HMBR when bound to Y-FAST (*k*_*1B*_ = (1.6 ± 0.2) × 10^−2^ min^−1^) and the respective probabilities p_1_ and (1–p_1_) of photodamaging the fluorogen and the protein tag (found to be 0.95 ± 0.01 and 0.05 ± 0.01, respectively). Taken together, these data demonstrate that fluorogen renewal in Y-FAST occurs with high efficiency as a result of a one order of magnitude lower photobleaching rate constant of the tag (*k*_*P*_) with respect to the photobleaching rate constant of the bound fluorogen (*k*_*1B*_). In sum, as long as the protein remains intact and provided that the fluorogen is in excess, damaged HMBR generally leaves the binding pocket and can be replaced with a new chromophore present in solution.

We next set out to understand the factors involved in the photobleaching of fluorogen-binding tags. Despite the benefit of fluorogen renewal, the protein tag ultimately undergoes a non-reversible alteration over time as shown by: (i) the partial fluorescence recovery upon fluorogen addition (Fig. 1C); and (ii) changes observed in the intrinsic fluorescence of aromatic amino acid residues within the protein scaffold (Figure S2). Various mechanisms can explain protein alteration. It is well documented for instance that one of the main pathways for photobleaching in fluorescent proteins is intertwined with molecular oxygen and photogenerated ROS^14–17^. For the Y-FAST:HMBR complex, removing oxygen from solution reduced the apparent photobleaching rate of both free- and bound-HMBR (Figure S3), indicating that ROS are produced and are likely one of the dominant photobleaching pathways in this fluorogenic system (for both protein and chromophore). Electrospray Ionization Mass Spectrometry (ESI-MS) top-down analysis of Y-FAST samples irradiated in excess of HMBR for 0, 4 or 8 hours showed a progressive increase in the overall mass of the protein in multiples of 16, in agreement with the addition of molecular oxygen by oxidation reactions (Figure S4). This ESI-MS top-down analysis also allowed us to exclude potential protein alterations involving covalent fluorogen crosslinking, as we observed no peaks indicating the formation of a covalent bond between the fluorogen and the protein scaffold.

**Figure 4:**
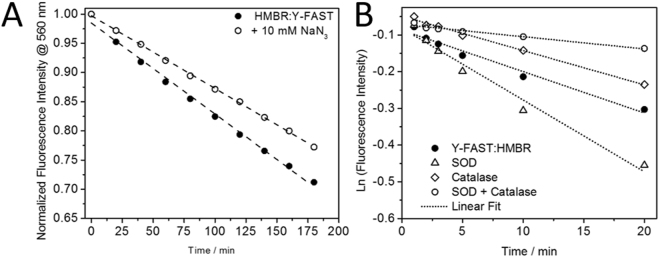
(**A**) Plot of the normalized fluorescence intensity of the HMBR:Y-FAST complex (15:3 µM) at 560 nm in the absence and presence of 10 mM sodium azide (NaN_3_), a physical quencher of singlet oxygen. Prolonged irradiation at 488 nm reveals a 2-fold decrease in the photodestruction of the protein, demonstrating the effect of singlet oxygen production by bound HMBR in the photobleaching of the Y-FAST:HMBR complex. (**B**) Plot of the linearized fluorescence intensity at 560 nm of Y-FAST:HMBR (15:3 µM) as a function of elapsed irradiation time (Ex 488 nm) in the presence of different reactive oxygen species quenchers (Catalase − 3.1 µM; Superoxide Dismutase − 3.3 µM), with the respective linear fits.

In order to obtain information on the photooxidation mechanisms, we irradiated the Y-FAST:HMBR complex in the presence of quenchers for different ROS: Catalase (quenching hydrogen peroxide)^31^, Superoxide Dismutase (SOD) (quenching superoxide ion)^31^, a mixture of Catalase and SOD, and NaN_3_^32^ (quenching singlet oxygen) (Fig. 4). Incubation of the Y-FAST:HMBR complex with catalase and SOD led to an almost 4-fold decrease of the photobleaching rate, catalase alone had a minor effect and SOD increased slightly the photobleaching rate. The reduction of photobleaching by a combined action of SOD and Catalase is in agreement with a bleaching mechanism involving superoxide radicals; SOD catalyses the partitioning of superoxide radicals in molecular oxygen and hydrogen peroxide, and Catalase subsequently transforms the generated hydrogen peroxide into molecular oxygen and water. On the other hand, the 2-fold decrease in the photobleaching rate when adding NaN_3_, a quencher of singlet oxygen, is in agreement with the additional involvement of singlet oxygen. These set of data show that the generation of superoxide radicals (likely *via* an electron transfer mechanism, *vide infra*) and of singlet oxygen (through triplet state quenching by molecular oxygen)^33^ both play a role in the photodestruction of Y-FAST. Singlet oxygen-driven oxidation was independently confirmed by the detection of kynurenin (a tryptophan oxidation product specific to singlet oxygen oxidation) both by optical signature (Figure S5 and SI Text 4) and by MS (*vide infra*)^34,35^. The use of hydroethidine (HE), a probe that converts to a red-shifted fluorescent product upon reaction with superoxide and hydrogen peroxide^36,37^, revealed that the production of these ROS by Y-FAST is comparable with EGFP or mVenus, but much lower (10-fold) than MiniSOG, a known ROS-producing protein^38–40^ (Figure S6).

**Figure 5:**
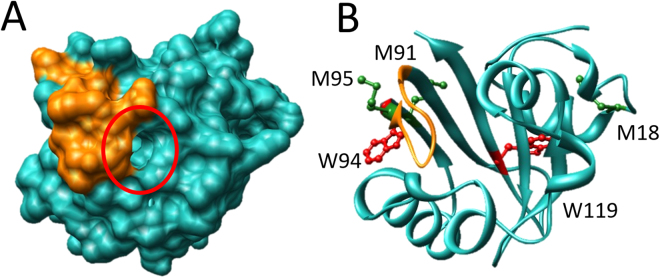
Illustration of Y-FAST (modelled using SWISS-PROT^51^ with PYP as a template - PDB ID: 2i9v^52^) with the randomized loop in Y-FAST^29^ depicted in orange and highlighting either: (**A**) the van der Waals surface, making visible the binding pocket (red circle); or (**B**) the secondary structure where key oxidized amino acids are highlighted as ball and stick (Methionines 18, 91 and 95 in green, Tryptophanes 94 and 119 in red).

In order to determine where Y-FAST was most affected by photooxidation we performed a combination of ESI-MS and trypsin-digested MS analysis on Y-FAST samples irradiated for 0, 4 and 8 hours (Figures S4, S7, S8 and SI Text 5). Conserved amino acid residues between PYP and Y-FAST buried inside the binding pocket^29,41,42^ remained unmodified. Methionine residues (specifically M18, M91 and M95) were mostly oxidized after 4 hours of irradiation, consistent with the high reactivity of these residues with ROS^43,44^. W94 and W119 were progressively oxidized over the 8 hours of irradiation (Fig. 5). Residues W94 and M95, localized in the loop originally modified for fluorogen binding during Y-FAST engineering^29^, were the most affected early on during the irradiation (confirmed by combined analysis of bottom-up and top-down MS experiments, see SI Text 5). Being W94 a conserved residue in all the clones initially selected during the development of Y-FAST^29^, its oxidation upon prolonged irradiation warranted further investigation. We mutated W94 to phenylalanine, mutant which showed lower binding affinity and dimmer fluorescence than Y-FAST (Figure S9), supporting that oxidation of W94 upon prolonged irradiation is likely responsible for a significant part of the fluorescence loss of the Y-FAST:HMBR complex.

From all the data combined, we can conclude that the Y-FAST:HMBR complex is photobleached by ROS formed upon irradiation of bound HMBR. Singlet oxygen is generated in a collision-dependent encounter of the triplet state of HMBR with molecular oxygen. In competition with this process, the formation of a radical species *via* electron-transfer from a nearby residue to the chromophore likely leads to the formation of superoxide and hydrogen peroxide. The latter is supported by literature data showing that phenolate moieties form radicals^45,46^ and the fact that ROS-generating fluorescent proteins display similar processes^38–40,47^. ROS generated upon illumination oxidize specific amino acid residues, namely tryptophan and methionine, leading to a loss of fluorescence and/or binding affinity which can be attributed to: (i) oxidation of amino acid residues away from the binding pocket leading to subtle changes in Y-FAST’s tertiary structure; and (ii) localized modifications in the evolved loop region leading to a loss of fluorescence and/or decrease in binding affinity. Admittedly the latter seems to bear a stronger influence in HMBR binding, which is consistent with the known diffusion of ROS away from the binding pocket^48^ and the reaction rate constants of these residues with ROS^44,49,50^.

## Conclusion

In this study, we developed an experimental and theoretical framework to study whether and how fluorogen renewal reduces the apparent photobleaching rate of fluorogen-binding tags using Y-FAST as a case study. Our approach is generic and should be applicable to any fluorogen-based systems to determine the degree and rate of alteration of both the fluorogen and the protein tag upon light irradiation. This approach allowed us to demonstrate that fluorogen renewal can significantly reduce the apparent photobleaching rate of Y-FAST. In addition, we presented a systematic analysis by optical spectroscopy and mass spectrometry of the factors reducing the photostability of this fluorogen-binding tag. In the case of Y-FAST, photostability is limited by protein photooxidation by ROS generated by the fluorogen upon irradiation. Our analysis suggests that Y-FAST can be optimized in two ways: (i) Specific amino acid residues could be mutated to reduce the photodamage caused to the protein or minimize the electron-donating properties of nearby amino acids;^42^ and (ii) chemical modifications could be introduced in the chromophore to reduce intersystem crossing and/or reduce the electron-withdrawing capacity of the deprotonated excited state of HMBR^8^. In conclusion, this study provides a general strategy to evaluate the photoresistance of fluorogen-based reporters, a much-needed standard for comparison of different fluorogen binding tags in their fluorogen renewal and usefulness in bioimaging.

## Materials and Methods

### Chemicals

4-hydroxy-3-methylbenzylidene-rhodanine (HMBR) was synthesized as previously described^29^. Sodium phosphate dibasic heptahydrate (≥98%), sodium phosphate monobasic anhydrous (≥96%), Imidazole (≥99%), Hydroethidine (HE) (≥95%), Catalase from bovine liver (≥70%, ≥10kU/mg), Superoxide Dismutase bovine (SOD) (≥90%, ≥2.5kU/mg), D_2_O (99.9%) and sodium azide (99.5%) were obtained from Sigma-Aldrich and used as received.

### Cloning, expression and purification of Y-FAST, Y-FAST^W94F^, MiniSOG, EGFP and mVenus

The plasmids pAG87, pAG136 et pAG137 for bacterial expression of Y-FAST, EGFP and mVenus were previously described^29^. The plasmid for bacterial expression of MiniSOG was obtained by inserting the sequence coding for MiniSOG in pET28 vector using *Nhe* I and *Xho* I restriction sites. The plasmid for bacterial expression of the mutant Y-FAST^W94F^ was generated by site directed mutagenesis from pAG87. Y-FAST, Y-FAST^W94F^, MiniSOG, EGFP and mVenus were expressed and purified following previously described protocols with small modifications^29^. Briefly, after elution from the Ni-NTA beads with a PBS solution supplemented with 0.5 M Imidazole, the purified protein fractions (as confirmed by SDS-PAGE) were buffer changed to PBS buffer (sodium phosphate 50 mM, NaCl 150 mM, pH 7.4, *I* = 280 mM) in PD-10 desalting columns (GE Healthcare) to remove excess imidazole.

### Instrumentation and Irradiation Methods

Steady state UV-Vis absorption spectra were recorded using a Cary 300 UV-Vis spectrometer (Agilent Technologies), equipped with a Versa20 Peltier-based temperature-controlled cuvette chamber (Quantum Northwest) and fluorescence data were recorded using a LPS 220 spectrofluorometer (PTI, Monmouth Junction, NJ), equipped with a TLC50TM Legacy/PTI Peltier-based temperature-controlled cuvette chamber (Quantum Northwest). For photobleaching, photoisomerization and probe experiments the samples were irradiated with a current-controlled LED, either a Luxeon Rebel (peak wavelength between 460–485 nm with a typical bandwidth of 20 nm) or a Luxeon Z Blue LXZ1-PB01 (peak wavelength between 460–480 nm with a typical bandwidth of 20 nm) (LUMILEDS), collimated and spectrally selected using an aspheric condenser (ACL2520, f = 20 mm) (Thorlabs) and a bandpass filter (HQ480/40 nm) (Chroma Technology Corp.), aligned in a 90° degree angle with respect to the photomultiplier detector of the spectrofluorometer. The incident power at the sample was measured using a Nova II power meter equipped with a PD300 (P/N7Z02410) photodiode (Ophir Photonics), where the sensor was partially covered with black tape to yield a 1 cm^2^ sensitive area. LED intensity changes during the course of the experiments were measured using a small Si photodiode (S1336-44BQ) placed directly underneath the cuvette chamber window and the fluorescence data normalized for measured power changes over time. O_2_ was removed by gaseous exchange of air by N_2_ in a sealed cuvette for over 30 min while the solution was under constant stirring. The needle blowing N_2_ was kept just above (and not inside) the liquid to avoid protein denaturation. All experiments were performed with 2 mL solutions in 1 cm^2^ quartz cuvettes with an irradiation window corresponding to 60% of the solution volume. To avoid any effect from diffusion stirring was kept constant throughout irradiation in all experiments. Affinity constants were determined by spectrofluorometric titration using a Spark 10 M plate reader (Tecan) (Ex 480 ± 10 nm, Em 560 ± 10 nm, 25 °C) using a 96 well-plate with 0.05 μM protein and increasing concentrations of HMBR.

### Reactive Oxygen Species Formation

For the hydroethidine (HE) experiments, all tested proteins were incubated with ~25 μM HE and irradiated using one of the LED previously described. The corresponding control experiments, i.e., Y-FAST with HE, HMBR with HE and HE alone showed a minor (less than 5%) formation of HE photoproducts due to autooxidation within the experimental time window.

### Mass Spectrometry

*Top-Down Approach:* Samples were acidified with formic acid (1% v:v final concentration), desalted and eluted with 70:30 acetonitrile: 1% of aqueous formic acid using a C18 Zip-Tip (Millipore-Merk, Darmstadt, Germany). Eluted samples were injected into a FT-ICR mass spectrometer (LTQ-FT Ultra, ThermoFisher Scientific, San Jose, CA, USA) equipped with a nano-electrospray (nESI) source. The MS1 spectra were acquired using the ICR analyser set at a resolution of 100 000. The MS/MS spectra were performed in collision induced fragmentation mode (CID) in the linear ion trap (collision energy: 35, isolation width: 10 m/z) and the generated fragments were analysed in the ICR cell set at a resolution of 100 000. The experimental spectra were deconvoluted with the Xtract tool of Xcalibur 2.1 (ThermoFisher Scientific). Deconvoluted MS/MS spectra were manually interpreted. *Bottom-up approach:* Samples were washed four times with a solution of 50 mM ammonium bicarbonate, pH 7.5, using Microcon-10KDa devices (Millipore-Merk, Darmstadt, Germany) to remove phosphate. In order to avoid sample losses, protein digestion was carried in 100 μL inside the filter devices adding 1.0 μg of trypsin (Sequencing grade, modified from bovine pancreas; Roche Diagnostics, Meylan, France) and incubated overnight at 37 °C while shaking. Cleaved peptides were filtered (10 min, 13 000 g, room temperature) and acidified with 10 μL of a 10% aqueous formic acid solution. 6 μL of peptide solution were injected on a capillary reversed-phase column (C_18_ Acclaim PepMap100, 75-μm i.d., 150-mm length, 10-nm particle size; Dionex-Thermo Scientific, Chelmsford, MA, USA), at a constant flow rate of 220 nL/min, with a gradient 2–40% buffer B in buffer A in 45 min (buffer A: water/acetonitrile/trifluoroacetic acid 98:2:0.1 (v/v/v), buffer B: water/acetonitrile/trifluoroacetic acid 10:90:0.1). Mass spectrometry was performed with the setup described above, using the Top 7 acquisition method (1 full scan MS at resolution 50 000; range of 400–2000 m/z), followed by 7 MS/MS (LTQ) on the 7 most intense peaks, with 90 s dynamic exclusion. Data were analysed with the software Peaks Studio (BSI, Waterloo, Canada) with a *de novo* sequencing approach (precursor Mass tolerance: 10 ppm, fragment Mass tolerance: 0.5 Da, variable modification; methionine oxidation, asparagine/glutamine de-amidation, enzyme: trypsin). Unspecified variable PTMs were found using PEAKS PTM algorithm checking for 485 modifications.

## Electronic supplementary material

Supporting Information
