# Supplementary material for: Chromophore Renewal and Fluorogen-Binding Tags: A Match Made to Last
Source: Sci Rep. 2017 Sep 26;7:12316. doi: 10.1038/s41598-017-12400-9 (PMC5615068; doi:10.1038/s41598-017-12400-9)
Supplement: Supplementary file 1 — Supporting Information [file 41598_2017_12400_MOESM1_ESM.pdf]

Supporting Information

Chromophore Renewal and Fluorogen-Binding Tags: A Match Made to Last

Frederico M. Pimenta,[1,2] Giovanni Chiappetta,[3] Thomas Le Saux,[1,2] Joëlle Vinh,[3] Ludovic Jullien,[1,2,*] Arnaud Gautier[1,2,*]

[1] *École Normale Supérieure, PSL Research University, UPMC Univ Paris 06, CNRS, Département de Chimie, PASTEUR, 24 rue Lhomond, 75005 Paris, France*

[2] *Sorbonne Universités, UPMC Univ Paris 06, ENS, CNRS, PASTEUR, 75005 Paris, France*

[3] *ESPCI Biological Mass Spectrometry and Proteomics USR 3149 CNRS/ESPCI ParisTech, Paris, France*

[*] *Corresponding authors:*

Arnaud Gautier (arnaud.gautier@ens.fr)
Ludovic Jullien (ludovic.jullien@ens.fr)

Contents:

SI Figures S1 – S9

SI Texts S1-S5

SI References

SUPPLEMENTARY FIGURES

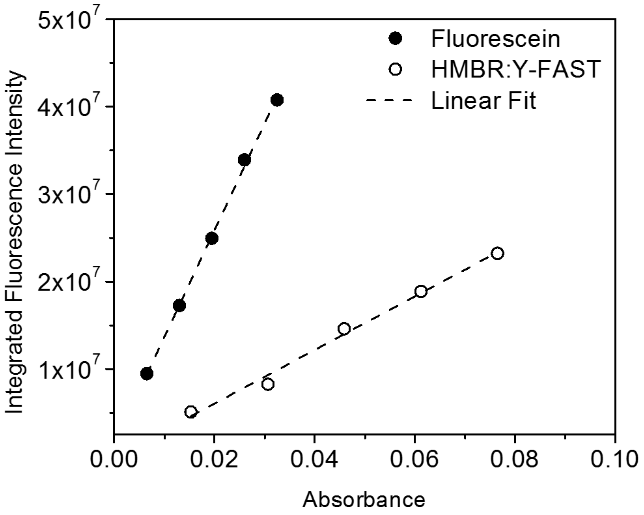

Figure S1. (A) Plot of the integrated fluorescence intensity at different concentrations for HMBR:Y-FAST solvated in PBS pH 7.4 and Fluorescein (0.1 M NaOH),[1] the latter used as fluorescence standard to determine the fluorescence quantum yield of the HMBR:Y-FAST complex. Samples were excited at 480 nm and absorbance kept below 0.05 to avoid inner-filter effects. The herein obtained quantum yield for HMBR:Y-FAST complex, ϕ_F = 0.23± 0.03, replaces the previously published number.

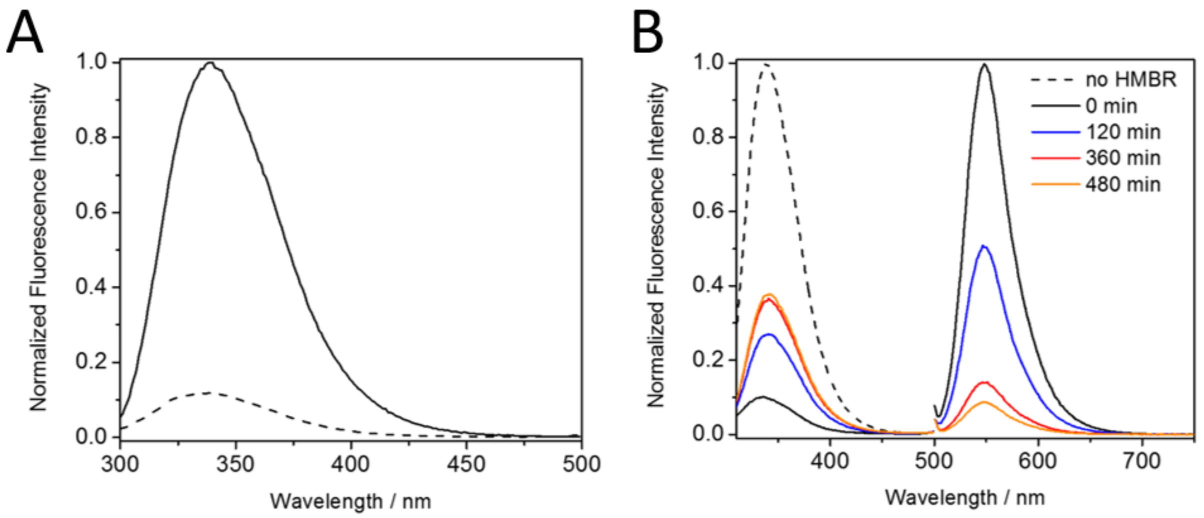

Figure S2. (A) Emission spectra of tyrosine and tryptophan residues either in the absence (solid line) or presence of HMBR (dashed line). The emission spectra was obtained with λ_{exc} = 280 nm using the same slit size to allow comparison of fluorescence intensity. The decrease in fluorescence intensity is consistent with an energy-transfer mechanism from the protein aromatic amino acid residues to the bound fluorogen. (B) Fluorescence spectra of aromatic amino acid residues (left series of spectra) and HMBR (right series of spectra) in an equimolar solution of HMBR:Y-FAST (3:3 μM) (~80% complex) recorded as a function of irradiation at 488 nm (243 W m^{-2}). While the fluorescence emission from bound HMBR continuously drops towards zero, the fluorescence emission of aromatic amino acids asymptotically increases towards a level which is significantly lower than its value in the absence of HMBR. This observation is consistent with protein damage as a consequence of HMBR irradiation over time.

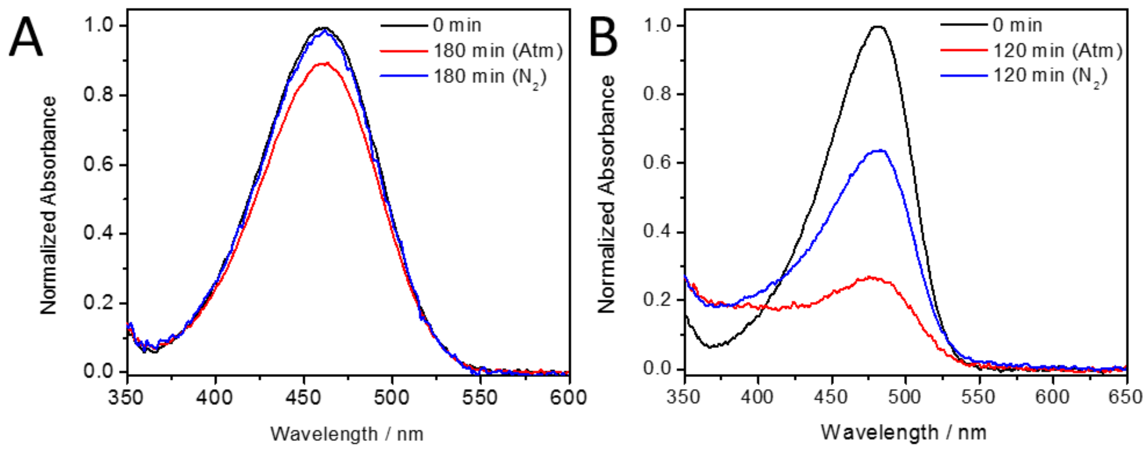

Figure S3. Plots of the absorption spectra of (A) deprotonated HMBR (pH 10) (3 μM) and (B) Y-FAST:HMBR (15:3 μM) complex upon irradiation at 488 nm under either atmosphere- or N_2-saturated conditions, demonstrating the role of molecular oxygen in the photodestruction of both HMBR and Y-FAST:HMBR. The observed photodamage in the HMBR:Y-FAST solution under N_2-saturation results from slow non-oxygen-dependent photodegradation pathways revealed by removing faster molecular oxygen-dependent photodegradation pathways.

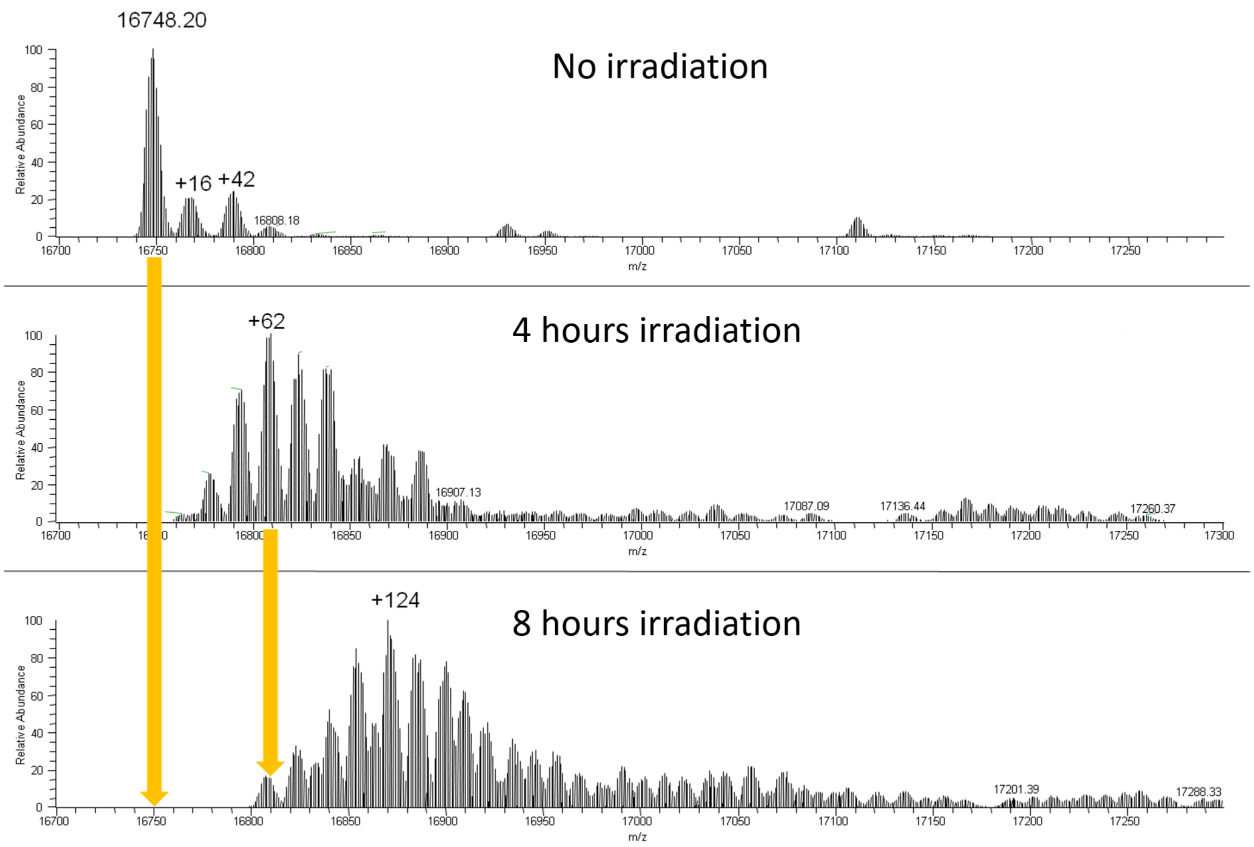

Figure S4. MS spectra of Y-FAST irradiated 0, 4 or 8 h at 488 nm with 243 W m^{-2} where HMBR was in excess (3:15 µM) with respect to Y-FAST. Solutions were up concentrated after irradiation. The initial peak observed at zero hours of irradiation (16 748.20 Da) matches the expected mass of Y-FAST with the attached His-Tag used for protein purification, i.e., un-modified Y-FAST is the major form before irradiation.

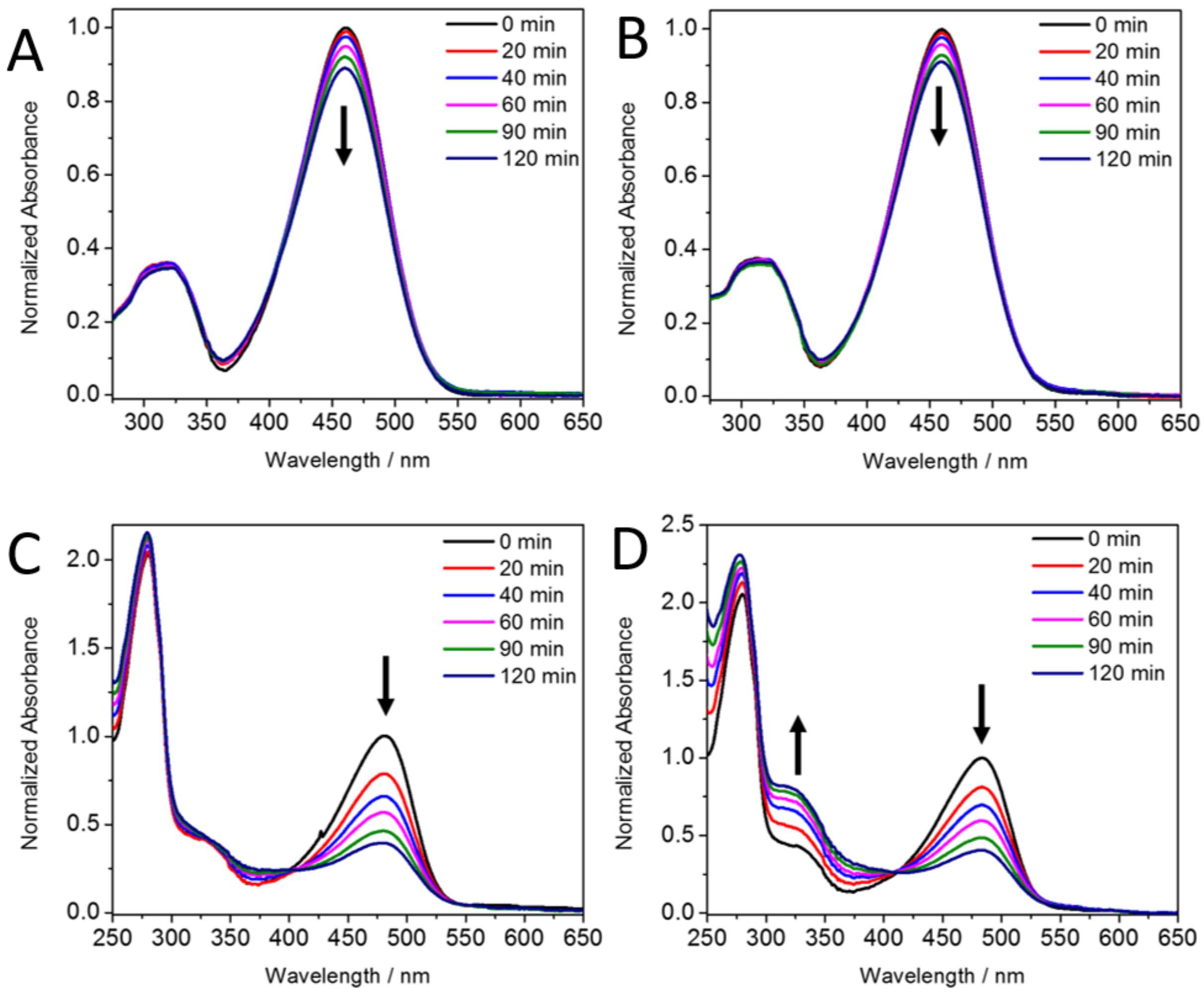

Figure S5. Absorption spectra of HMBR (A and B) or the Y-FAST:HMBR (15:3 μM) complex (C and D) recorded as a function of elapsed irradiation time (Ex 488 nm). The samples were dissolved in either an H_2O-buffered solution at pH 10.0 (A) or pH 7.4 (C), or in D_2O-buffered solution at pD 10.4 (B) or pD 7.8 (recall that pD = pH + 0.4).

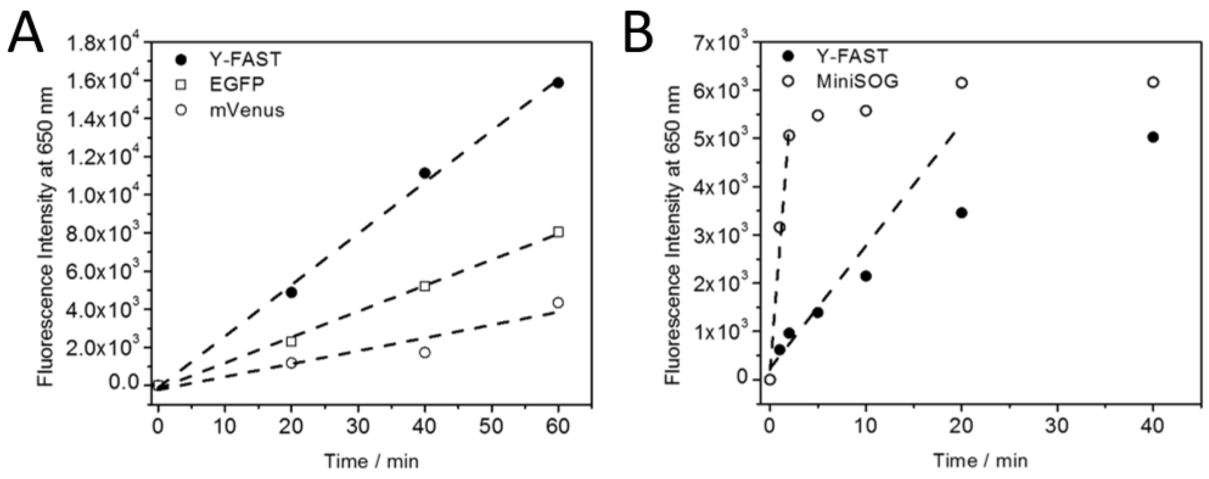

Figure S6. (A) Plot of the fluorescence intensity at 650 nm of the hydroethidine (HE) oxidation product generated upon irradiation of (A) EGFP, mVenus and Y-FAST:HMBR and (B) Y-FAST:HMBR and MiniSOG (Exc 488 nm). Data were normalized for differences in absorption at the irradiation wavelength. HE was in large excess at initial times and protein concentration for B were significantly higher than in A, justifying the saturation curve displayed at long times. Dashed lines correspond to linear fits to the data, either (A) full dataset or (B) at initial time points (3 first points for MiniSOG, 4 first points for Y-FAST).

A

B

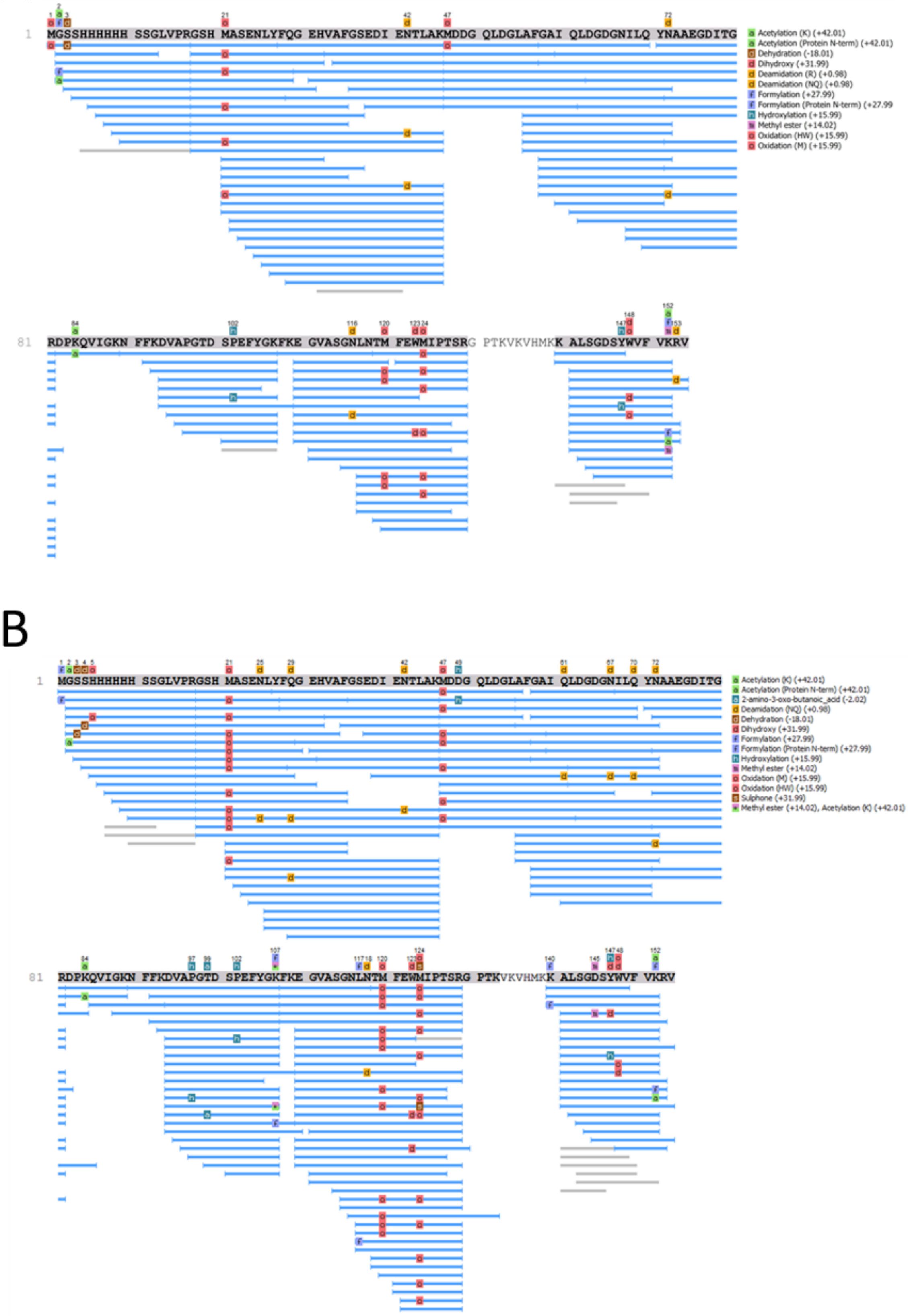
1 MGSSHHHHHH SSGLVPRGSH MASENLYFQG EHVAFGSEDI ENTLAKMDDG QLDGLAFGAI QLDGDGNILQ YNAAEGDITG
81 RDPKQVIGKN FFKDVAPGTD SPEFYGKFKE GVASGNLNTM FEWMIPTSRG PTKVKVHMKK ALSGDSYWVF VKRV
Acetylation (K) (+42.01)
Acetylation (Protein N-term) (+42.01)
Dehydration (-18.01)
Dihydroxy (+31.99)
Deamidation (R) (+0.98)
Deamidation (NQ) (+0.98)
Formylation (+27.99)
Formylation (Protein N-term) (+27.99
Hydroxylation (+15.99)
Methyl ester (+14.02)
Oxidation (HW) (+15.99)
Oxidation (M) (+15.99)
1 MGSSHHHHHH SSGLVPRGSH MASENLYFQG EHVAFGSEDI ENTLAKMDDG QLDGLAFGAI QLDGDGNILQ YNAAEGDITG
81 RDPKQVIGKN FFKDVAPGTD SPEFYGKFKE GVASGNLNTM FEWMIPTSRG PTKVKVHMKK ALSGDSYWVF VKRV
Acetylation (K) (+42.01)
Acetylation (Protein N-term) (+42.01)
2-amino-3-oxo-butanoic_acid (-2.02)
Deamidation (NQ) (+0.98)
Dehydration (-18.01)
Dihydroxy (+31.99)
Formylation (+27.99)
Formylation (Protein N-term) (+27.99)
Hydroxylation (+15.99)
Methyl ester (+14.02)
Oxidation (M) (+15.99)
Oxidation (HW) (+15.99)
Sulphone (+31.99)
Methyl ester (+14.02), Acetylation (K) (+42.01)

C

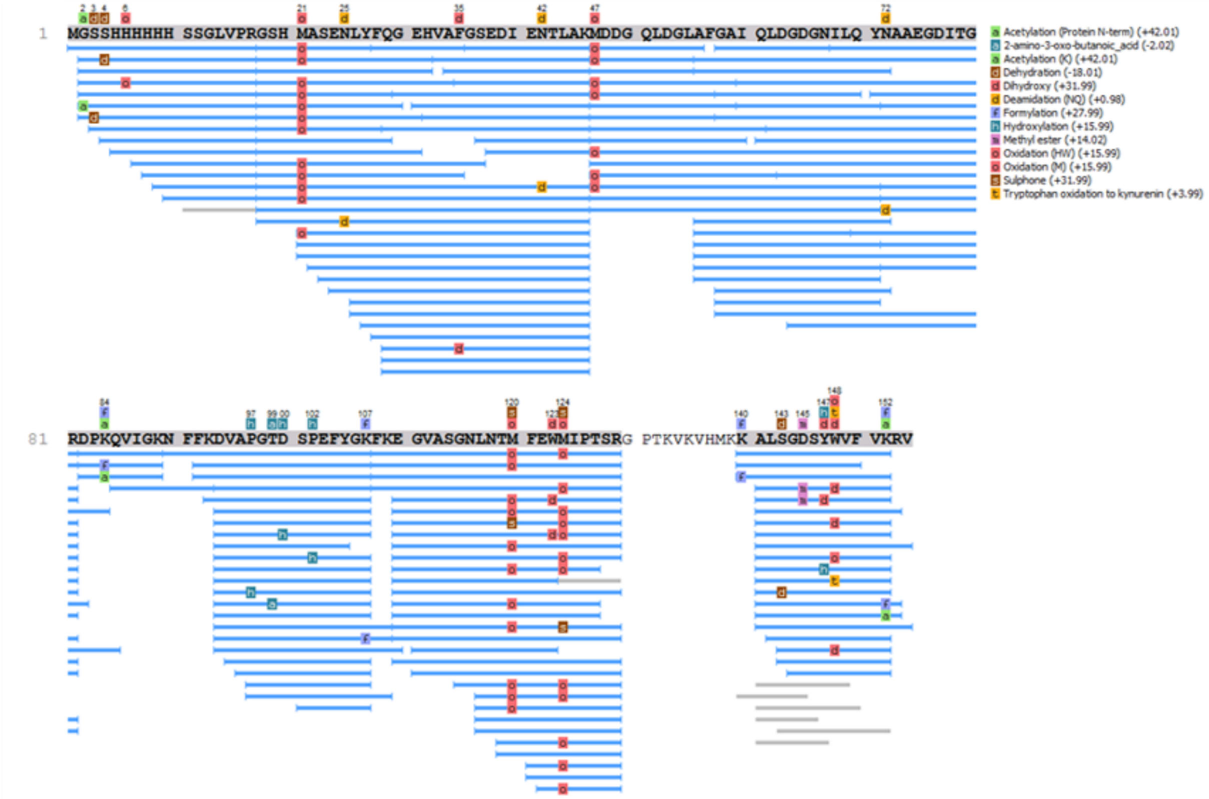

Figure S7. Sequence coverage obtained by the bottom-up analysis of Y-FAST irradiated 0 h (A), 4 h (B) or 8 h (C) at 488 nm with 243 W m^{-2}, where HMBR was in excess with respect to Y-FAST (3:15 µM). Solutions were up concentrated after irradiation. Each blue line corresponds to an assigned MS/MS spectrum covering the selected sequence region, and relevant Post-Translational Modifications (PTM) are reported. Sequence numbering corresponds to Y-FAST with a His-tag (+ 29 amino acids).

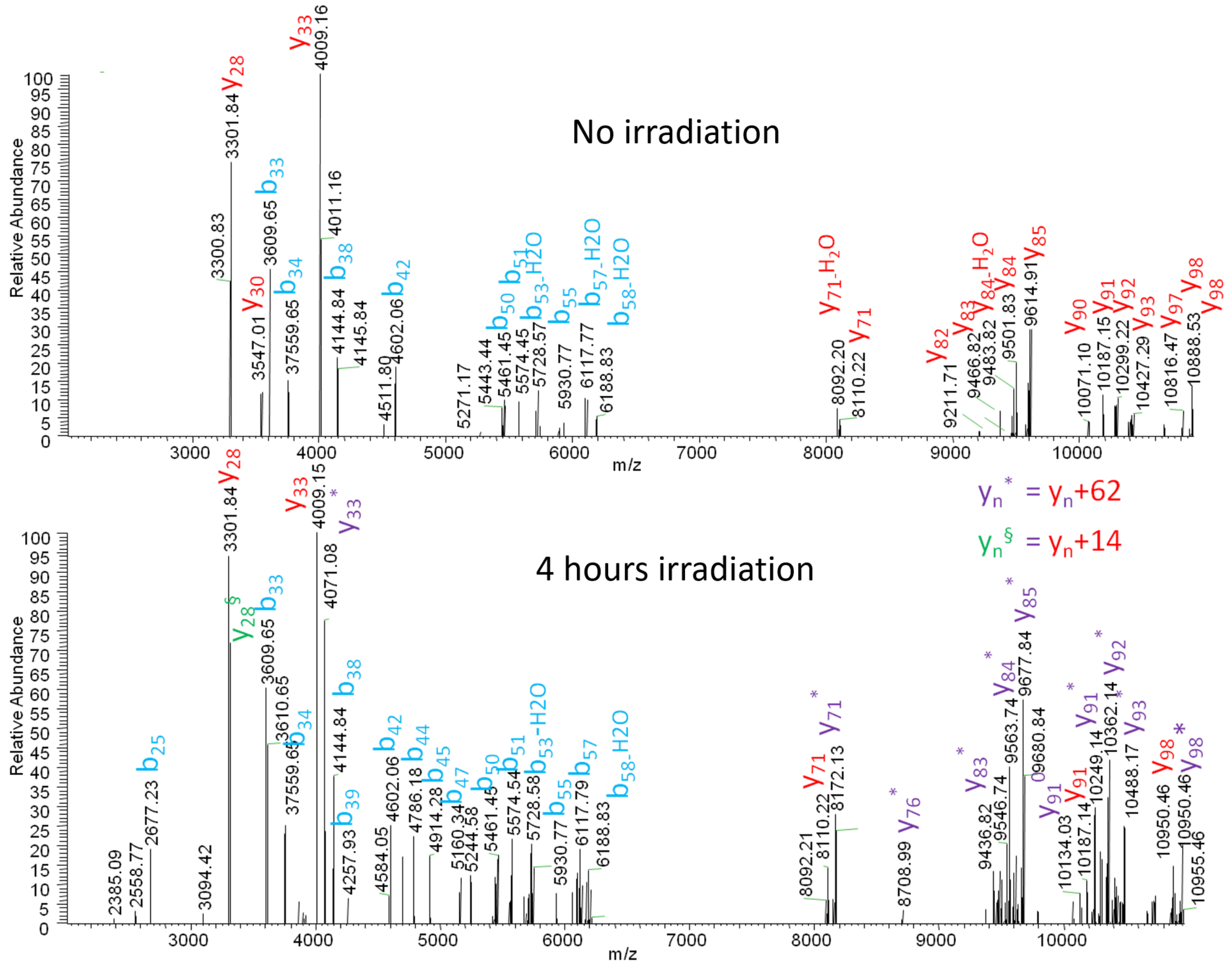

Figure S8. MS/MS spectra of intact wild type Y-FAST and Y-FAST iso-form +62Da observed after 4 hours of irradiation at 488 nm with 243 W m^{-2} where HMBR was in excess with respect to Y-FAST (3:15 µM). Solutions were up concentrated after irradiation. The Y-FAST spectrum after 4 hours of irradiation (see Figure S4) is characterized by the presence of two parallel y-ion series, at +14 Da and +62 with respect to the natural Y-FAST y-ion series.

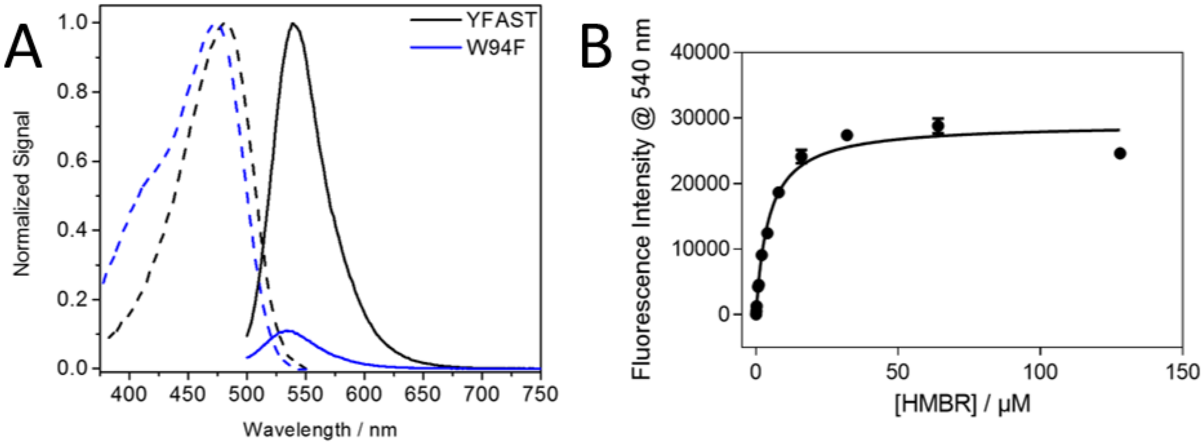

Figure S9. (A) Absorption (dashed lines) and emission (solid lines) spectra of HMBR:Y-FAST (black lines) and HMBR: Y-FAST^{W94F} (blue lines) at 2:40 µM ratio of fluorogen:protein. The fluorescence emission spectra were normalized for differences in complex formation. The shoulder in the absorption at ~400 nm and lower fluorescence quantum yield observed in the Y-FAST^{W94F} mutant demonstrates the lower binding affinity of HMBR for this mutant. (B) Steady-state HMBR titration of Y-FAST^{W94F} mutant, where the fluorescence intensity of the protein:fluorogen complex at 560 nm is measured at a fixed concentration of protein (0.05 µM) and increasing concentrations of HMBR (n = 3). A non-linear fit (I_F=F_{max} [HMBR]/(K_D + [HMBR])) of the data (corrected for free HMBR emission at each concentration) yields a dissociation constant (K_D) of 4.5 ± 0.42 µM. This demonstrated the reduced affinity of HMBR to this mutant, and by extent to oxidative modifications to this particular amino acid residue (W94) that occur during long-term irradiation, as observed by both MS and optical experiments.

SUPPLEMENTARY TEXTS

SI Text 1: Fluorescence Quantum Yield Determination (Figure S1)

The quantum yield of fluorescence (Φ_F) for the HMBR:Y-FAST complex was determined using different concentrations of HMBR and a constant concentration of Y-FAST, such that the complex formed was always ~100% (Figure S1). Complex absorbance was kept below 0.05 and plotted as a function of the integrated emission intensity according to the equation:

$$\Phi_F^S = \Phi_F^R \cdot \frac{I_S}{I_R} \cdot \frac{Abs_R}{Abs_S} \cdot \left(\frac{n^S}{n^R}\right)^2$$

where *S* and *R* are the sample and reference, respectively, *I* is the integrated intensity of fluorescence emission, *Abs* is the absorbance at the excitation wavelength and *n* is the refractive index of each solution.[2] Fluorescein (in 0.1 M NaOH) was used as a standard (Φ_F = 0.92± 0.2).[1] The quantum yield of Φ_F = 0.23 ± 0.03 obtained herein replaces the value previously published.[3]

SI Text 2: Photoreversible reactions (Figure 2)

HMBR shares structural similarities with the chromophores of GFP and PYP, both known to undergo a reversible dark state photoconversion upon illumination.[4,5] Continuous illumination at low light intensity (71 W m^{-2}) showed that HMBR undergoes a similar reversible photoconversion, both in the absence and the presence of an excess of Y-FAST, ensuring complete complex formation (Figure 2). For the Y-FAST:HMBR complex, a small reversible loss of fluorescence was observed (~ 8% of the total fluorescence signal) with an associated relaxation time of 1.1 s and a thermal return of 1.5 s (Figure 2B). Free deprotonated HMBR at pH 10.5 showed a similar photoconversion (Figure 2A). Irradiation of Y-FAST:HMBR at 488 nm for 12 hours (ceasing irradiation for 5 min every hour) indicated that this reversible photoconversion to a dark state plays a minor role in the overall loss of fluorescence, which is seemingly dominated by non-reversible processes (Figure 2C, D).

SI Text 3: Theoretical Models, Photoisomerization and Photobleaching Data Analysis

We consider a protein scaffold **P** (total concentration P_{tot}) reacting with a fluorogen **1F** to yield a bound state **1B** (total concentration F_{tot}) according to the reaction:

$$\mathbf{P} + \mathbf{1F} \leftrightarrows \mathbf{1B} \qquad (1)$$

associated with the forward and backward reaction rates, k_1 and k_{-1}, and the association constant K_1. Here we analyse the change in concentration of these species over time when the system is closed and illuminated, assuming both reversible (on a short time scale) and irreversible (on a long time scale) photochemical reactions.

Reversible photochemical reactions

At a short time scale, we consider that both **1F** and **1B** can reversibly photoisomerize to **2F** and **2B**, respectively (Scheme S1).

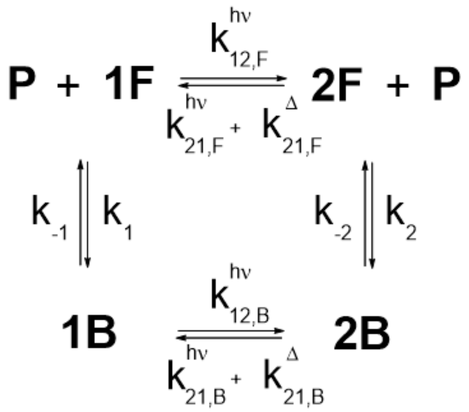

Scheme S1. Overall scheme governing the dynamics of the system on a short time scale.

k_2 and k_{-2} are the forward and backward rate constants involved in the complexation of the reversibly photoisomerized **2F** state with **P,** yielding **2B**. $k_{12,X}^{h\nu}$, $k_{21,X}^{h\nu}$, and $k_{21,X}^{\Delta}$ designate the rate constants for the photoisomerization (superscript $h\nu$) and thermal isomerization (superscript Δ) in the directions **1X→2X** and **2X→1X** of the state **X** (where **X** = **F** or **B**).

We assume that the exchange between **P**, **1F**, and **1B**, and between **P**, **2F**, and **2B** are fast at the short time scale of the reversible photochemical reactions,* allowing us to reduce the overall scheme shown above to:

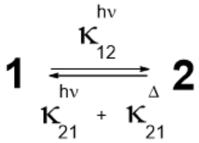

Scheme S2. Reduced dynamic scheme governing the dynamics of the system on a short time scale.

, where **1** and **2** are the virtual states associated to $1=1F+1B$ and $2=2F+2B$ (with $1+2=F_{tot}$), and where $\kappa_{12}^{h\nu}$, $\kappa_{21}^{h\nu}$, and κ_{21}^{Δ} are the reduced rate constants driving the apparent exchange between the states **1** and **2**. By assuming that the protein scaffold is in excess with respect to the fluorogen (Figure 2), one can then express the concentrations of **1F**, **1B**, **2F**, and **2B**, and the three reduced rate constants $\kappa_{12}^{h\nu}$, $\kappa_{21}^{h\nu}$, and κ_{21}^{Δ} as a function of the rate constants in scheme S1 (Eq. 2-8).

* The relaxation time associated with the fluorogen complexation by Y-FAST at room temperature lies in the 0.1 s range when the concentrations of the free and bound states are equal.[3] This order of magnitude is significantly lower than the second time scale for photoisomerization (Figure 2) and the hour time scale for photobleaching (Figure 3).

$$[1F] = \frac{1}{1+K_1 P_{tot}}[1] \tag{2}$$

$$[1B] = \frac{K_1 P_{tot}}{1+K_1 P_{tot}}[1] \tag{3}$$

$$[2F] = \frac{1}{1+K_2 P_{tot}}[2] \tag{4}$$

$$[2B] = \frac{K_2 P_{tot}}{1+K_2 P_{tot}}[2] \tag{5}$$

$$\kappa_{12}^{h\nu} = \frac{1}{1+K_1 P_{tot}} k_{12,F}^{h\nu} + \frac{K_1 P_{tot}}{1+K_1 P_{tot}} k_{12,B}^{h\nu} \tag{6}$$

$$\kappa_{21}^{h\nu} = \frac{1}{1+K_2 P_{tot}} k_{21,F}^{h\nu} + \frac{K_2 P_{tot}}{1+K_2 P_{tot}} k_{21,B}^{h\nu} \tag{7}$$

$$\kappa_{21}^{\Delta} = \frac{1}{1+K_2 P_{tot}} k_{21,F}^{\Delta} + \frac{K_2 P_{tot}}{1+K_2 P_{tot}} k_{21,B}^{\Delta} \tag{8}$$

The concentration of **1** and **2** vary over time according to the differential equation: (9)

$$-\frac{d[1]}{dt} = \frac{d[2]}{dt} = \kappa_{12}^{h\nu}[1] - \left(\kappa_{21}^{h\nu} + \kappa_{21}^{\Delta}\right)[2] \tag{9}$$

, which yields

$$[1]^{\infty} - [1] = [2] - [2]^{\infty} = -[2]^{\infty} \exp\left(-\frac{t}{\tau_{12}}\right) \tag{10}$$

, where

$$[1]^{\infty} = \frac{\kappa_{21}^{h\nu} + \kappa_{21}^{\Delta}}{\kappa_{12}^{h\nu} + \kappa_{21}^{h\nu} + \kappa_{21}^{\Delta}} \mathrm{F}_{tot} \tag{11}$$

$$[2]^{\infty} = \frac{\kappa_{12}^{h\nu}}{\kappa_{12}^{h\nu} + \kappa_{21}^{h\nu} + \kappa_{21}^{\Delta}} \mathrm{F}_{tot} \tag{12}$$

$$\tau_{12} = \frac{1}{\kappa_{12}^{h\nu} + \kappa_{21}^{h\nu} + \kappa_{21}^{\Delta}} \tag{13}$$

Under illumination (71 Wm^{-2} at 488 nm), τ_{12} is described by Eq. 13. In the absence of the scaffold, τ_{12} = 0.9 ± 0.05 s (close to the temporal resolution of our experimental setup), which yields $\kappa_{12}^{h\nu} + \kappa_{21}^{h\nu} \gtrsim 0.89 \pm 0.09$ s^{-1} (Figure 2A). In the presence of the scaffold, τ_{12} = 1.1 ± 0.08 s, which yields $\kappa_{12}^{h\nu} + \kappa_{21}^{h\nu} \gtrsim 0.24 \pm 0.15$ s^{-1} (Figure 2B). Considering that the molar absorption coefficient of the protein: fluorogen complex is significantly larger than the one of the free fluorogen in the buffered aqueous solution at the considered wavelength, this observation suggests that fluorogen photoisomerization is hindered upon protein encapsulation.

In the absence of illumination, Eqs. 2-5 and 10 predict a single exponential decay for the fluorescence intensity, with a relaxation time τ_{12}:

$$\tau_{12} = \frac{1}{\kappa_{21}^{\Delta}} \tag{14}$$

In the absence of the scaffold, τ_{12} = 4.5 ± 1.2 s, $\kappa_{21}^{\Delta} = k_{21,F}^{\Delta}$ =0.22 ± 0.06 s^{-1} (Figure 2A), while in the presence of the scaffold τ_{12} = 1.5 ± 0.3 s, κ_{21}^{Δ} =0.67 ± 0.13 s^{-1} (Figure 2B). This indicates that the rate constant for the thermally-driven back fluorogen isomerization is larger within the protein cavity than in the aqueous solution ($k_{21,B}^{\Delta} > k_{21,F}^{\Delta}$).

Irreversible photochemical reactions

We now consider that all species in Scheme S1 (**1F**, **1B**, **2F**, **2B)** can encounter irreversible photochemical reactions, being described by their respective photobleaching rate constants (k_{1F}, k_{1B}, k_{2F}, and k_{2B}). In the specific cases of **1B** and **2B** we additionally consider that photobleaching can either lead to the destruction of **1F** or **P**, and **2F** or **P** (to yield **1F*** and **P***, and **2F*** and **P*** respectively) with the respective rate constants $p_1 k_{1B}$ and $(1-p_1)k_{1B}$, and $p_2 k_{2B}$ and $(1-p_2)k_{2B}$ (Scheme S3).

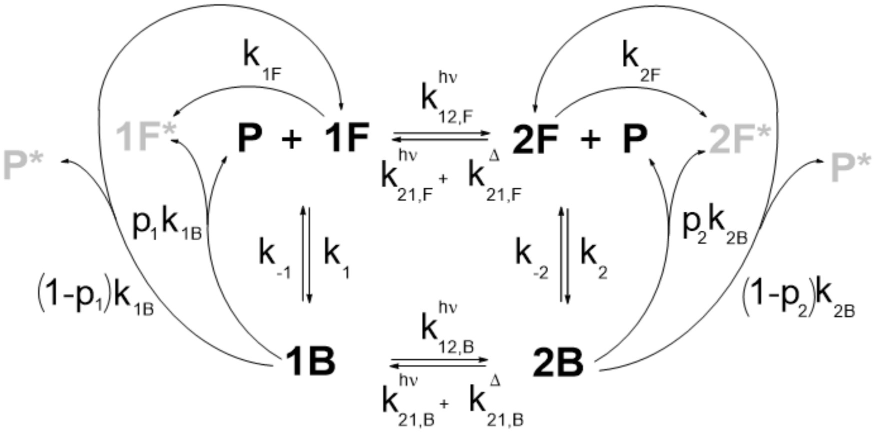

Scheme S3. Overall scheme governing the dynamics of the system on a long time scale.

Beyond the short time scale investigated in the preceding section, it is relevant to consider that the Y-FAST scaffold **P** and the virtual species **F=1+2** are involved in their respective annihilation reactions:

$$\mathbf{P} \rightarrow \mathbf{P^*} \tag{15}$$

$$\mathbf{F} \rightarrow \mathbf{F^*} \tag{16}$$

, with the associated rate constants k_P and k_F. We introduce

$$K_{12} = \frac{\kappa_{12}^{h\nu}}{\kappa_{21}^{h\nu}+\kappa_{21}^{\Delta}} \tag{17}$$

and

$$K = \frac{1}{1+K_{12}}K_1 + \frac{K_{12}}{1+K_{12}}K_2 \tag{18}$$

Assuming that $F_{tot} \gg P_{tot}$ (such that the concentrations in the bound states **1B** and **2B** are lower than the corresponding free states **1F** and **2F**) and that F_{tot} is large enough to secure that $KF_{tot} \gg 1$ (as evidenced by the formation of the bound states in the absorption spectrum displayed in Figure 3E), one can derive:

$$P_{tot} = P_{tot}^0 \exp[-k_P t] \quad (19)$$

, with
$$k_P = \frac{1}{\tau_P} = (1-p_1)\frac{K_1}{K}\frac{1}{1+K_{12}}k_{1B} + (1-p_2)\frac{K_2}{K}\frac{K_{12}}{1+K_{12}}k_{2B} \quad (20)$$

, and
$$F_{tot} = (F_{tot}^0 - \alpha P_{tot}^0)\exp(-k_{\mathrm{F}}t) + \alpha P_{tot}^0 \exp(-k_{\mathrm{P}}t) \quad (21)$$

, with
$$k_{\mathrm{F}} = \frac{1}{\tau_F} = \frac{1}{1+K_{12}}k_{1F} + \frac{K_{12}}{1+K_{12}}k_{2F} \quad (22)$$

, and
$$\alpha = \left(p_1\frac{K_1}{K}\frac{1}{1+K_{12}}k_{1B} + p_2\frac{K_2}{K}\frac{K_{12}}{1+K_{12}}k_{2B}\right)\frac{\tau_P\tau_F}{\tau_F-\tau_P} \quad (23)$$

, where F_{tot}^0 and P_{tot}^0 designate the initial concentration of fluorogen and protein scaffold.

The concentration of **1F**, **1B**, **2F**, **2B**, and **P** is then given by:

$$[1F] \approx \frac{1}{1+K_{12}}\mathrm{F}_{tot} \quad (24)$$

$$[1B] \approx \frac{K_1}{K}\frac{1}{1+K_{12}}P_{tot} \quad (25)$$

$$[2F] \approx \frac{K_{12}}{1+K_{12}}\mathrm{F}_{tot} \quad (26)$$

$$[2B] \approx \frac{K_2}{K}\frac{K_{12}}{1+K_{12}}P_{tot} \quad (27)$$

Two observables have been experimentally measured to extract kinetic information: the absorbance $A(t)$ and the fluorescence intensity $I_F(t)$ originating from solutions containing **1F**, **2F**, **1B**, and **2B**. These observables have been measured after relaxation of the reversible photoisomerization observed at short time scale such that the expressions of the absorbance and the fluorescence intensity can be described by:

$$A(t) = [\varepsilon_{1F}F_{tot} + \varepsilon_{1B}P_{tot}]l \quad (28)$$

$$I_F(t) = Q_{1F}F_{tot} + Q_{1B}P_{tot} \quad (29)$$

, where ε_{1F}, ε_{1B}, l, Q_{1F}, and Q_{1B} respectively designate the molar absorption coefficients of **1F** and **1B**, the light pathway, and the brightness of **1F** and **1B**.

From Eq. 21, for $P_{tot} = 0$ the fluorogen photobleaching is described by a single exponential with the relaxation time τ_F. Under illumination (324 W.m^{-2} at 488 nm) and in the absence of the scaffold **P,** we observed a slow linear decay of the fluorescence emission (expected at short times for a slow exponential decay) and extracted $k_{\mathrm{F}} = (4.7 \pm 0.8) \times 10^{-5}$ min^{-}

[1], which we subsequently adopted for the apparent photobleaching rate of **F** in the absence of the scaffold **P** (Figure 3A and B).

Under illumination (324 W.m^{-2} at 488 nm) and in the presence of the scaffold **P** (under 10 fold excess of **F**, large excess as assumed in the model), we recorded both changes in absorbance (specifically at 405 nm and at 480 nm) and fluorescence emission at 560 nm as a function of irradiation. From Eq. 19, 21 and 24-27, the latter is expected to exhibit a mono exponential decay with a relaxation time of $1/k_P$, which is observed on our experimental data (Figure 3D), where τ_P =1012 ± 33 min, k_P =(9.9 ± 0.3) × 10^{-4} min^{-1}. The absorbance decays at 405 and 480 nm are expected to be described by a sum of two exponentials with the relaxation times $1/k_F$ and $1/k_P$ given in Eq. 20 and 22. The experimental data however decays mono exponentially at both 405 nm and 480 nm. This observation originates from the large difference between k_F and k_P so that $\exp(-k_\mathrm{F}t) \approx 1$ in Eq. 21 in the considered time window. By assuming $K_2 = 0$ (in relation to a poorer fit of the photoisomerized fluorogen when bound to Y-FAST), that all fluorogen destruction occurs from interaction with the protein and the absorbance change over time is then described by:

$$\frac{A(t)}{A(0)} = \frac{\varepsilon_{1F}\left(F_{tot}^0 - \alpha P_{tot}^0\right)}{\varepsilon_{1F}F_{tot}^0 + \varepsilon_{1B}P_{tot}^0} + \frac{(\alpha\varepsilon_{1F} + \varepsilon_{1B})P_{tot}^0}{\varepsilon_{1F}F_{tot}^0 + \varepsilon_{1B}P_{tot}^0}\exp(-k_P t) \tag{30}$$

$$\alpha = (p_1 k_{1B})\tau_P \tag{31}$$

$$k_P = (1 - p_1)k_{1B} \tag{32}$$

The pre-exponential factor of the exponential term on Eq. 30, obtained from the absorbance mono exponential decay (Figure 3F), the protein (P_{tot}^0) and fluorogen (F_{tot}^0) concentrations, $\varepsilon_{1F}^{405\,nm}$ = 34 000 M^{-1} cm^{-1} and $\varepsilon_{1B}^{405\,nm}$ = 10 000 M^{-1} cm^{-1} were used to determine α. This latter term was then used with Eq. 31 and 32 (derived from 23 and 20 respectively), to retrieve p_1 = 0.95 ± 0.01 and $k_{1\mathrm{B}}$ = (1.6 ± 0.2) × 10^{-2} min^{-1}. Errors of 10% were assumed for the molar absorption coefficients.

SI Text 4: Interpretation of the H_2O/D_2O experiment (Figure S5)

The isotope effect is routinely used to study the presence and effect of singlet oxygen, whose lifetime increases by ~20 fold, from ~3.5 µs to ~67 µs in water upon deuteration.[6–8] The photobleaching rate of the free solvated chromophore at basic pH did not change with buffer deuteration, indicating the absence of chemical quenching of singlet oxygen by the anionic chromophore. Photobleaching in the presence of Y-FAST (3 µM of HMBR to 15 µM of Y-FAST) showed that even though the absorbance and fluorescence decrease of HMBR was independent of deuteration, an absorption band centered at ~325 nm became more pronounced in deuterated PBS. Irradiation of that band revealed a fluorescence signal independent of HMBR (free or bound) or the aromatic residues present in the protein, pointing to the formation of N-formylkynurenine, a tryptophan derivative formed by singlet oxygen oxidation.[9–11]

These data indicate that: (i) a triplet excited state of HMBR is formed upon irradiation when the latter is bound to the protein; (ii) singlet oxygen is generated by this system upon

irradiation, as demonstrated by the pronounced formation of a characteristic product of its reaction with tryptophan in deuterated water; and (iii) singlet oxygen seems to play a minor role in the chromophore photodestruction when free in solution. All the former agree well with the incomplete recovery of tryptophan and tyrosine emission upon irradiation (Figure S2B). Alternatively, other ROS generated via a radical-mediated mechanism can also modify these aromatic residues.

SI Text 5: Mass Spectrometry Data Analysis (Figures S4, S7, S8)

Y-FAST samples (containing a His-Tag used for protein purification), either not irradiated or irradiated for 4 or 8 hours at 488 nm with 243 W m^{-2} (HMBR in excess (5:1) with respect to Y-FAST during irradiation) were first analysed by a bottom-up approach by trypsin digestion followed by LC-MS/MS analysis as described in the Material and Methods. Collected data were processed using a *de novo* sequencing approach by PEAKS PTM algorithm (PEAKS STUDIO), identifying the presence of up to 485 Post-Translational Modifications (PTM) with no *a priori* knowledge. For clarity, numbering on residues will follow the original Y-FAST sequence (including the initiating methionine) and exclude the His-Tag (which adds 29 residues to the protein sequence).

The top-down approach on the non-irradiated Y-FAST sample (Figure S4 top spectrum) reveals that the un-modified Y-FAST is the major form before irradiation. Other modified forms present in the same MS spectrum, such as mono-oxidized (+16 Da), acetylated non-oxidized and acetylated-oxidized (+58 Da), correspond to 20% or less of unmodified Y-FAST (formylated or methyl esterified forms were not detected in this MS spectrum). We obtained a 94% sequence coverage for non-irradiated Y-FAST by bottom-up (Figure S7A), where some oxidative modifications are already observed, such as methionine oxidations (M18, M91, M95), proline hydroxylation (P73), tryptophan oxidation (W119), tryptophan di-hydroxylations (W94, W119) and non-oxidative acetylations (N-terminal, K55, K123). Other secondary modifications such as formylation (+28 Da; N-terminal, K123) and methyl esterification (+14 Da; K123) were also detected.

A 96% of protein sequence coverage was obtained by the bottom up analysis of the Y-FAST sample irradiated for 4 hours (Figure S7B), where we observed an overall increase in the number of oxidative modifications. The number of assigned MS/MS spectra increased for M18, M91 and M95 (oxidative sites previously detected in non-irradiated Y-FAST, see Figure S7A and B) which could be correlated to an increased extent of the oxidation. Other modified sites appear, oxidation of P68, T70, Y118, sulphonation of M95 and methyl-esterification of D116. A top-down analysis on this sample (Figure S4 middle spectrum) showed a Gaussian distribution of multi-oxidized forms (up to 7 oxygen atoms added) with a maximum corresponding to a +62 Da form (a small percentage of protein presents one modification corresponding to a methyl-esterification). The precursor ion corresponding to the +62 Da form (m/z=842.1, z=20) was isolated in the linear ion trap and submitted to CID fragmentation (Figure S8 top spectrum). The same experiment was performed on non-irradiated Y-FAST (precursor ion m/z 838.3, z=20) as a reference spectrum (Figure S8 bottom spectrum). The MS/MS spectra interpretation showed that

the +62 Da form is unique (instead of a mixture of isobaric randomly-oxidized forms) and it corresponds to the addition of three oxygen atoms (positioned between the residues F92 and I96) and one methyl group (positioned between the protein C-Terminal and the residue P97). Crossing the top-down data with the bottom-up data indicates that the major Y-FAST iso-form after 4h of irradiation is characterized by the di-hydroxylation of W94, oxidation of M95 and methyl-esterification of D116.

A 94% of protein coverage in the bottom up analysis of Y-FAST irradiated for 8 hours demonstrated an increase on the extent of oxidation with respect to 4 hours of irradiation (Figure S7C and B, respectively). New modifications observed are the sulphonation of M91, di-hydroxylation of F6, hydroxylation of D71, the β-elimination of S114 and the conversion of W119 to kynurenin. The top-down confirmed the increase of the extent of oxidation, with the most abundant iso-form being the +124 Da form (corresponding to the double of the modification observed after 4 hours) and the major iso-form after 4 hours being the least abundant (Figure S4 bottom spectrum). Unfortunately, the complexity of the MS spectrum of this sample precludes a deeper analysis as done for previous samples due to the quality of the MS/MS spectra.

SI References

1. Magde, D., Wong, R. & Seybold, P. G. Fluorescence quantum yields and their relation to lifetimes of rhodamine 6G and fluorescein in nine solvents: improved absolute standards for quantum yields. *Photochem. Photobiol.* **75,** 327–334 (2002).
2. Lakowicz, J. R. *Principles of Fluorescence Spectroscopy*. (Springer US, 2006).
3. Plamont, M.-A. *et al.* Small fluorescence-activating and absorption-shifting tag for tunable protein imaging in vivo. *Proc. Natl. Acad. Sci. USA* **113,** 497–502 (2016).
4. Remington, S. J. Fluorescent proteins: maturation, photochemistry and photophysics. *Curr. Opin. Struct. Biol.* **16,** 714–721 (2006).
5. Jung, G., Wiehler, J. & Zumbusch, A. The photophysics of green fluorescent protein: influence of the key amino acids at positions 65, 203, and 222. *Biophys. J.* **88,** 1932–1947 (2005).
6. Schweitzer, C. & Schmidt, R. Physical mechanisms of generation and deactivation of singlet oxygen. *Chem. Rev.* **103,** 1685–1757 (2003).
7. Wilkinson, F., Helman, W. & Ross, A. Rate constants for the decay and reactions of the lowest electronically excited singlet state of molecular oxygen in solution. An expanded and revised compilation. *J. Phys. Chem. Ref. Data* **24,** 663–1021 (1995).
8. Ogilby, P. R. Singlet oxygen: there is indeed something new under the sun. *Chem. Soc. Rev.* **39,** 3181–3209 (2010).
9. Walrant, P. & Santus, R. N-formyl-kynurenine, a tryptophan photooxidation product, as a photodynamic sensitizer. *Photochem. Photobiol.* **19,** 411–417 (1974).
10. Fukunaga, Y., Katsuragi, Y., Izumi, T. & Sakiyama, F. Fluorescence characteristics of kynurenine and N'-formylkynurenine. Their use as reporters of the environment of tryptophan 62 in hen egg-white lysozyme. *J. Biochem.* **92,** 129–141 (1982).
11. Davies, M. J. Reactive species formed on proteins exposed to singlet oxygen. *Photochem. Photobiol. Sci.* **3,** 17–25 (2004).
